# Damage assessment in structures using artificial neural network working and a hybrid stochastic optimization

**DOI:** 10.1038/s41598-022-09126-8

**Published:** 2022-03-23

**Authors:** H. Tran-Ngoc, S. Khatir, T. Le-Xuan, H. Tran-Viet, G. De Roeck, T. Bui-Tien, M. Abdel Wahab

**Affiliations:** 1 Laboratory Soete, Department of Electrical Energy, Metals, Mechanical Constructions, and Systems, Faculty of Engineering and Architecture, Ghent University, 9000 Gent, Belgium; 2 Department of Bridge and Tunnel Engineering, Faculty of Civil Engineering, University of Transport and Communications, Hanoi, Vietnam; 3 Faculty of Civil Engineering, Ho Chi Minh City Open University, Ho Chi Minh City, Vietnam; 4 Department of Civil Engineering, KU Leuven, 3001 Leuven, Belgium; 5 Faculty of Mechanical, Electrical and Computer Engineering, School of Engineering and Technology, Van Lang University, Ho Chi Minh City, Vietnam

**Keywords:** Civil engineering, Computational science, Software

## Abstract

Artificial neural network (ANN) has been commonly used to deal with many problems. However, since this algorithm applies backpropagation algorithms based on gradient descent (GD) technique to look for the best solution, the network may face major risks of being entrapped in local minima. To overcome those drawbacks of ANN, in this work, we propose a novel ANN working parallel with metaheuristic algorithms (MAs) to train the network. The core idea is that first, (1) GD is applied to increase the convergence speed. (2) If the network is stuck in local minima, the capacity of the global search technique of MAs is employed. (3) After escaping from local minima, the GD technique is applied again. This process is applied until the target is achieved. Additionally, to increase the efficiency of the global search capacity, a hybrid of particle swarm optimization and genetic algorithm (PSOGA) is employed. The effectiveness of ANNPSOGA is assessed using both numerical models and measurement. The results demonstrate that ANNPSOGA provides higher accuracy than traditional ANN, PSO, and other hybrid ANNs (even a higher level of noise is employed) and also considerably decreases calculational cost compared with PSO.

## Introduction

During service life, engineering structures can be damaged by a wide variety of impacts such as corrosion, overload, environmental factors, natural disasters, and human impacts. Regular inspections and health monitoring are essential to find and repair any defect occurring in structures^1^. This plays an important role in increasing the lifetime and operational effectiveness of the structure. During the past decades, numerous research approaches have been applied for structural health monitoring (SHM)^2–4^.

ANN is an intelligent computational technique inspired by the way that the human biological system employs to process data. With recent ground-breaking advances, ANN has been applied commonly to deal with complicated issues in different fields during the past decades^5–10^. However, it is acknowledged that ANN still has its fundamental drawbacks. To look for the best solution, ANN employs BP algorithms based on GD methods using the principle of a downward slope. This makes the network get stuck when falling into concave surfaces (valleys). Hence, the obtained result is only the local best instead of the global best. This causes a reduction in the accuracy and efficiency of ANN.

With the global search capacity of optimization algorithms^11–15^, in recent years, many researchers have also provided workable solutions to overcome the local minima drawbacks and improve the efficiency of ANN. For instance, Samir et al.^17^ proposed PSO to enhance training parameters (weight and bias) of ANN to detect damages in a laminated composite. In their research, PSO was employed to determine optimal starting points. This approach probably assisted the network in avoiding initial local minima. Nevertheless, because PSO was only applied to identify training parameters of the first steps, the network may be still trapped in other local bests in the subsequent steps. With the same approach, Rajendra et al.^18^ employed genetic algorithm (GA) to improve the effectiveness of ANN, when predicting optimized parameters for biodiesel production. In the work of Yazdanmehr et al.^19^, GA was coupled with ANN to find the optimal material producing nanocrystalline powder with minimum coercion. A new approach combining GA with ANN (GAANN) to evaluate and optimize production is proposed in the research of Azadeh et al.^20^.

It is easily seen that the above-mentioned approaches^17–20^ and other current approaches only applied solutions to local minimum prevention by choosing a beneficial starting position employing the global search capability of other algorithms. Although the substantial benefits of the aforementioned proposed methods to ANN are indisputable, it is commonly acknowledged that looking for a good starting point remains a challenge. Specifically, choosing a good starting point only may assist the network in avoiding the first local minimum (the first valley). However, a network often has many local bests widely distributed, especially if the network contains a complex error surface. Hence, the solution of selecting good starting points may no further be beneficial because the particles of the network may be still trapped in subsequent local minima (other valleys) in the process of seeking the best solutions.

To remedy these shortcomings, in this paper, we propose a novel ANN working parallel with metaheuristic algorithms, which surpasses previous approaches^17–20^ that only apply the capacity of global search techniques to look for good starting points for the network. The core idea of this approach is that the global search capacity is applied to work parallel with the GD technique to prevent the network from trapping in the locally optimal solutions throughout the process of seeking the best solution instead of only choosing a beneficial starting position used in previous approaches. Last but not least, in this research, to enhance the effectiveness of global search techniques, we employ a hybrid PSOGA to determine training parameters during the process of training the network (weight and bias). Specifically, GA with crossover and mutation operators is applied to generate the initial elite populations, and those populations are then employed to seek the best solution based on the global search capacity of PSO.

To consider the operational condition of structures in the real application, the effect of noise is also considered and the results show that ANNPSOGA surpasses ANN as well as other hybrid-ANNs regarding accuracy even it employs a higher level of noise for input data. To validate the effectiveness of the proposed method, ANNPSOGA is compared with PSO, traditional ANN and approaches mentioned in Refs.^17–20^ named as ANNPSO. The efficiency and applicability of ANNPSOGA are evaluated by employing both numerical and measured models with different damage scenarios of the tested structures (single and multiple damages). We conduct all calculation tasks in the computer with processor: Intel (R) core™ i7-8650U; CPU @ 1.9 GHz. For all examples in this work, Levenberg–Marquardt (LM) backpropagation algorithm is used to train the network.

The main contributions of this work are depicted as follows:

- A hybrid algorithm combining PSO and GA is proposed to enhance the capacity of global search of traditional PSO and GA.
- A parallel working between ANN and a hybrid algorithm (GAPSO) is developed to deal with local minimum problems of ANN in the most radical way.
- The effectiveness and correctness of the proposed method are proved through both numerical model and measurement in which each model contains a large number of scenarios (single damages, multiple damages).
- A comparison between the proposed method and numerous other algorithms including traditional PSO, traditional ANN, and other hybrid ANNs is conducted.
- The proposed approach also compares the effect of noise on the trained data with traditional ANN, MA, and other hybrid ANNs.

## Methodology

ANN is an intelligent computational program imitating the method that the human biological system employs to process data, commonly applied to SHM over the past decades. Nevertheless, since ANN applies BP algorithms using GD-based learning techniques to look for the best solution, this may pose a considerable risk of being entrapped in local minima to the network, especially, when the network generates too numerous locally optimal solutions.

**Figure 1:**
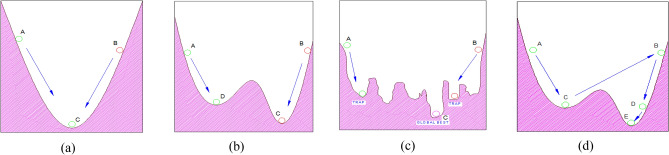
The process of determining the best solution using GD techniques of ANN (**a**) one local minimum, (**b**) less local minima, (**c**) many local minima; (**d**) proposed approach.

The performance of ANN depends crucially on whether the network is entrapped in the local bests or not. A typical example (Fig. 1) depicts the process of seeking the best solution of ANN using the GD technique. In Fig. 1a, when the network solely contains the best solution, even though the departure position is (A) or (B), global solution (C) will be surely determined. In Fig. 1b, the network creates complex error surfaces with two local bests. If the network departs from (B), the global solution (C) is also determined correctly. However, if the starting position is from (A), the network only probably determines the local best (D) instead of the global best (C). Therefore, choosing an advantageous departure point may only help the network to avoid local minima for some simple problems.

It is recognized that many researchers have adopted this approach to address the local minimum problems of ANN^17–20^. However, this approach is only applicable to simple cases when the network only has small numbers of the best locally optimal solutions or these local minima are only distributed at one side. For complicated issues e.g. SHM problems, the network contains many local minima located everywhere (as shown in Fig. 1c). Hence, the solution for selecting the beneficial departure points may no further be effective.

Therefore, it is necessary to come up with workable solutions when the network is entrapped in the best local solutions instead of relying on the fortune of possessing an advantageous departure point. In this paper, we propose applying the stochastic search capacity of PSOGA to prevent the network from being entrapped in the local bests throughout the process of looking for the global best. Figure 1d illustrates the use of PSOGA to overcome local minimum problems of ANN.

In Fig. 1d, if the starting point is from A, the GD technique will make the network fall into local best (D). In this case, to assist the network in escaping from the local best (D), PSOGA is applied. PSOGA uses stochastic search techniques based on natural phenomena that may assist the particles in escaping from local minima.

### ANNPSOGA

**Figure 2:**
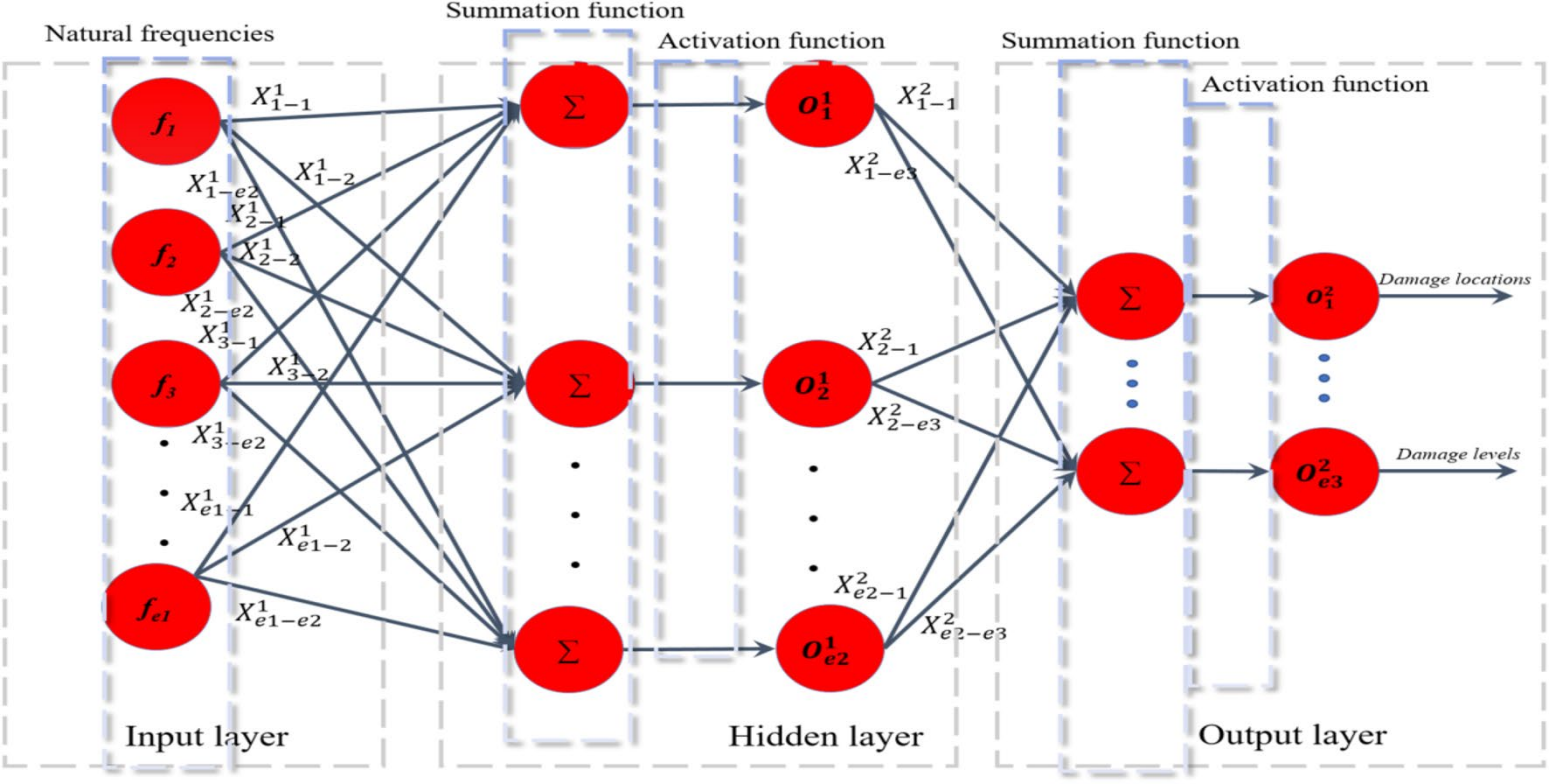
The network structure.

In this part, the methodology of ANNPSOGA is elucidated. First, data set from the input layer together with weight ratios, bias ratios are summed and relayed to the hidden layer (see Fig. 2) as follows:

1 $$\sum_{{z_{2} }}^{1} = \mathop \sum \limits_{{z_{1} , z_{2} }}^{{e_{1} ,e_{2} }} {\varvec{X}}_{{z_{1} z_{2} }} *f_{{z_{1} }} + {\varvec{X}}_{{z_{2} }} ;z_{1} \div \left( {1:e_{1} } \right), z_{2} \div \left( {1:e_{2} } \right)$$

where ${f}_{{z}_{1}}$ is data of the *z*_1_th element transferring from the input data to the hidden layer; ${\sum }_{{z}_{2}}^{1}$ denotes the input data of the *z*_2_th element of the hidden layer; ${X}_{{z}_{1}{z}_{2}}$, and ${X}_{{z}_{2}}$ indicate training parameters connecting the input layer and the hidden layer. The parameters ${e}_{1}$ and ${e}_{2}$ denote the number of elements of the input layer and the hidden layer, respectively. After that, a sigmoid function is employed to calculate outputs ${(O}_{{z}_{2}})$ at the hidden layer.

2 $${O}_{{z}_{2}}=\frac{1}{1+{e}^{- {\sum }_{{z}_{2}}^{1}}}$$

Elements of the hidden layer are then transferred to the output layer. This process applies Eq. (3).

3 $$\sum_{{z_{3} }}^{2} = \mathop \sum \limits_{{z_{2} , z_{3} }}^{{e_{2} , e_{3} }} {\varvec{X}}_{{z_{2} z_{3} }} *O_{{z_{2} }} + {\varvec{X}}_{{z_{3} }} ; z_{3} = \left( {1:e_{3} } \right)$$

${\sum }_{{z}_{3}}^{2}$ is the input of the *z*_3_th neuron of the output layer; ${e}_{3}$ indicates the number of neurons in the output layer.

4 $${O}_{{z}_{3}}=\frac{1}{1+{e}^{{-\sum }_{{z}_{3}}^{2}}}$$

${O}_{{z}_{3}}$ is the output at the output layer.

The deviation between predicted and real outputs $({O}_{{z}_{3}}^{k} \mathrm{and} {\overline{O} }_{{z}_{3}}^{k})$ is calculated.

5 $${\mathcal{E}}\left( {\varvec{X}} \right) = \mathop \sum \limits_{k = 1}^{{N_{0} }} \frac{1}{2}\frac{{\left( {O_{{z_{3} }}^{k} - \overline{O}_{{z_{3} }}^{k} } \right)^{2} }}{{N_{0} }}$$

${N}_{0}$ denotes the proportion of output data.

If the network is entrapped in the locally optimal solution, it means that the deviation between predicted and real outputs of the next step is not smaller than that of the previous one (shown in Eq. (6)).

6 $${\mathcal{E}}^{{{\varvec{t}} + 1}} \left( {\varvec{X}} \right)\, \nless \,{\mathcal{E}}^{{\varvec{t}}} \left( {\varvec{X}} \right)$$

$t$ is *t*th iteration.

In this case, PSOGA is employed to escape the network from local minima. Specifically, PSOGA is used to determine the new weight and bias coefficient (${\varvec{X}})$ for the network. Weight and bias values from the ANN are then organized as a single vector, which is used as initial parameters of particles of PSOGA:

7 $${{\varvec{Y}}}^{0}=\left[{{\varvec{y}}}_{1}^{0}, {{\varvec{y}}}_{2}^{0},\cdots , {{\varvec{y}}}_{n}^{0}\right],$$

8 $${{\varvec{y}}}_{{\varvec{i}}}^{0}={\left[{{\varvec{y}}}_{1i}^{0}, {{\varvec{y}}}_{2i}^{0},\cdots , {{\varvec{y}}}_{mi}^{0}\right]}^{T},\boldsymbol{ }i=1, 2, \dots , n.$$

where ${{\varvec{X}}}^{0}$ is weight and bias values from the ANN; *n* indicates the proportion of training parameters, *m* indicates the proportion of population, and *T* denotes a transposed matrix.

Calculating the values of ${\mathcal{E}}\left( {\varvec{Y}} \right)$ of initial populations based on Eq. (5).

9 $${\mathcal{E}}\left( {{\varvec{Y}}^{0} } \right) = \left[ {\begin{array}{*{20}c} {{\mathcal{E}}\left( {{\varvec{y}}_{1}^{0} } \right)} \\ {\begin{array}{*{20}c} {{\mathcal{E}}\left( {{\varvec{y}}_{2}^{0} } \right)} \\ {{\mathcal{E}}\left( {{\varvec{y}}_{3}^{0} } \right)} \\ {\begin{array}{*{20}c} . \\ . \\ \end{array} } \\ \end{array} } \\ {\begin{array}{*{20}c} . \\ {{\mathcal{E}}\left( {{\varvec{y}}_{n}^{0} } \right)} \\ \end{array} } \\ \end{array} } \right]_{n*1}$$

The particles with better quality are selected for crossover and mutation.

10 $${n}_{c}={\alpha }_{c}*n$$

${\alpha }_{c}$ is crossover percentage (${\alpha }_{c}$ = 0.8).

11 $${n}_{m}={\alpha }_{m}*n$$

${\alpha }_{m}$ is mutation percentage (${\alpha }_{m}$ = 0.1).

High quality obtained from GA is used to look for the best solution of PSO.

12 $${{\varvec{X}}}^{0}=\left[{x}_{1}^{0},{x}_{2}^{0},\dots ,{x}_{n}^{0}\right]$$

13 $${x}_{i}^{0}=[{{x}_{1i}}^{0},{{x}_{2i}}^{0},...,{{x}_{mi}}^{0}{]}^{T},i=\mathrm{1,2},...,n.$$

${x}_{1}^{0},{x}_{2}^{0},\dots ,{x}_{n}^{0}$ are initial population of PSO determined from GA.

14 $${{\varvec{V}}}^{0}=\left[{v}_{1}^{0},{v}_{2}^{0},\dots ,{v}_{n}^{0}\right]$$

15 $${v}_{i}^{0}=[{{v}_{1i}}^{0},{{v}_{2i}}^{0},...,{{v}_{mi}}^{0}{]}^{T},i=\mathrm{1,2},...,n.$$

${{\varvec{V}}}^{0}$ is for initial velocity of elements.

16 $${{\varvec{P}}}^{0}=\left[{p}_{1}^{0},{p}_{2}^{0},\dots ,{p}_{n}^{0}\right]$$

17 $${p}_{i}^{0}=[{{p}_{1i}}^{0},{{p}_{2i}}^{0},...,{{p}_{mi}}^{0}{]}^{T},i=\mathrm{1,2},...,n.$$

${{\varvec{P}}}^{0}$ is initially local optimal solution of elements.

18 $${{\varvec{G}}}^{0}=[{G}^{0}{]}_{m*1}$$

${{\varvec{G}}}^{0}$ is an initially global optimal solution of elements.

The limitation to the search space (${{\varvec{X}}}_{lower}, { {\varvec{X}}}_{upper})$ is applied.

19 $${{\varvec{X}}}_{lower}={\left[{{\varvec{X}}}_{min}\right]}_{m*n}$$

20 $${{\varvec{X}}}_{upper}={\left[{{\varvec{X}}}_{max}\right]}_{m*n}$$

Updating new position and velocity of particles at the 1th iteration

21 $${\varvec{V}}^{1} = w^{\prime}{\varvec{V}}^{0} + C^{\prime}r^{\prime}\left( {{\varvec{P}}^{0} - {\varvec{X}}^{0} } \right) + C^{\prime\prime}r^{\prime\prime}\left( {{\varvec{G}}^{0} - {\varvec{X}}^{0} } \right)$$

$C^{\prime}$ and $C^{\prime\prime}$ denote the learning factors;$w^{\prime}$ is the inertia weight parameter; $r^{\prime}$ and $r^{\prime\prime}$ are random values (0, 1);

22 $${{\varvec{X}}}^{1}={{\varvec{X}}}^{0}+{{\varvec{V}}}^{1}$$

23 $$\mathrm{If }{{\varvec{X}}}^{1}>{ {\varvec{X}}}_{upper}$$

24 $${{\varvec{X}}}^{1}={{\varvec{X}}}_{lower}+{rand*{\varvec{X}}}_{lower}$$

25 $$\mathrm{If }{{\varvec{X}}}^{1}<{ {\varvec{X}}}_{lower}$$

26 $${{\varvec{X}}}^{1}={{\varvec{X}}}_{lower}+{rand*{\varvec{X}}}_{lower}$$

Applying the objective function of Eq. (5) again.

Calculating ${\mathcal{E}}\left({\varvec{X}}\right)$ of particles at 1th iteration

27 $${{\mathcal{E}}\left({{\varvec{X}}}^{1}\right)=\left[{\mathcal{E}}\left({{\varvec{X}}}^{1}\right)\right]}_{m*1}$$

The best local solution (${{\varvec{P}}}^{1}$) and the best global solution (${{\varvec{G}}}^{1}$) of particles at 1th iteration are identified.

Updating new properties of particles at *i*th iteration

28 $${\varvec{V}}^{{\varvec{i}}} = w^{\prime}{\varvec{V}}^{i - 1} + C^{\prime}r^{\prime}\left( {{\varvec{P}}^{i - 1} - {\varvec{X}}^{i - 1} } \right) + C^{\prime\prime}r^{\prime\prime}\left( {{\varvec{G}}^{i - 1} - {\varvec{X}}^{i - 1} } \right)$$

29 $${{\varvec{X}}}^{i}={{\varvec{X}}}^{i-1}+{{\varvec{V}}}^{i}$$

30 $$\mathrm{If }{{\varvec{X}}}^{i}>{ {\varvec{X}}}_{upper}$$

31 $${{\varvec{X}}}^{i}={{\varvec{X}}}_{lower}+{rand*{\varvec{X}}}_{lower}$$

32 $$\mathrm{If }{{\varvec{X}}}^{i}<{ {\varvec{X}}}_{lower}$$

33 $${{\varvec{X}}}^{i}={{\varvec{X}}}_{lower}+{rand*{\varvec{X}}}_{lower}$$

Calculating ${\mathcal{E}}\left({\varvec{X}}\right)$ of particles at *i*th iteration

34 $${\mathcal{E}}\left({{\varvec{X}}}^{i}\right)=\left[{\mathcal{E}}\left({{\varvec{X}}}^{i}\right)\right]_{m*1}$$

Selecting the best local solution (${{\varvec{P}}}^{\mathrm{i}},$) and the best global solution (${{\varvec{G}}}^{\mathrm{i}}$) of particles at *i*th iteration.

35 $$If {\mathcal{E}}\left({{\varvec{X}}}^{i+1}\right)<{\mathcal{E}}\left({{\varvec{X}}}^{i}\right)$$

36 $${\mathcal{E}}\left({{\varvec{X}}}^{i+1}\right)={\mathcal{E}}\left({{\varvec{X}}}^{i+1}\right)$$

37 $$Otherwise {\mathcal{E}}\left({{\varvec{X}}}^{i+1}\right)= {\mathcal{E}}\left({{\varvec{X}}}^{i}\right)$$

The search process finishes and the best solutions are achieved.

38 $${\mathcal{E}}\left({\varvec{G}},k\right)=min {\mathcal{E}}\left({\varvec{X}}\right)$$

39 $${\varvec{G}}={{\varvec{X}}}^{{\varvec{q}}}$$

*q* is *q*th iteration, *q* ∈ [*0, N*].

**Figure 3:**
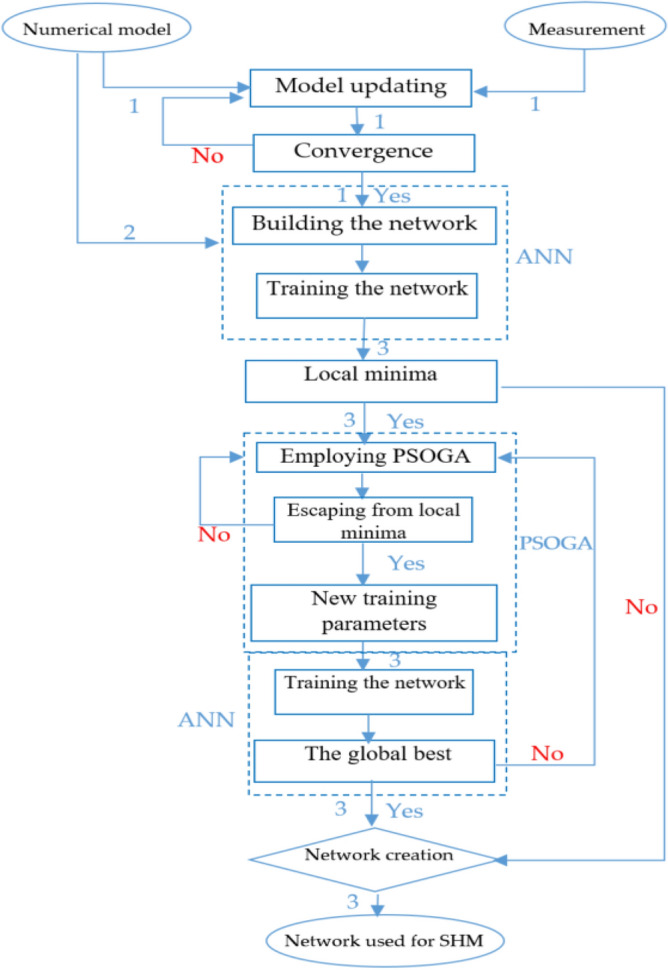
The diagram of the proposed approach.

The best solutions (training parameters) are determined and converted to weight and bias values $\left({\varvec{X}}\right)$ of ANN used to train the network. The methodology of ANNPSOGA is elucidated in Fig. 3.The diagram of the proposed approach.

Figure 3 represents the 2 models used in this paper, which are summarised as follows.

**Case 1**: the numerical model is applied. The network building process is conducted without the model updating process (the diagram follows the arrows 2 and 3).

**Case 2**: The proposed method is used to detect damages for a steel beam in the laboratory with different damage scenarios (the diagram follows the arrow 1 and 3). To convert the damaged properties ($$D)$$ of the beams from measurements into output data of the network, the following equations are applied^21^.

40 $$D = \left( {1 - \frac{{E_{1} }}{{E_{0} }}} \right)*100\%$$

where ${E}_{0}\mathrm{ and} {E}_{1}$ are intact and damaged stiffness.

## Numerical examples

**Figure 4:**
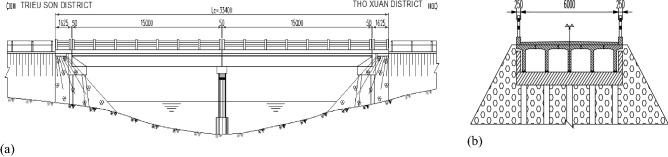
(**a**) The layout of the bridge and (**b**) cross-section of the bridge.

Sy bridge (Fig. 4) is a simply supported reinforced concrete beam bridge connecting Trieu son district and Tho Xuan district in Thanh Hoa province in the North of Vietnam. The bridge was built and has been operating since 1995. The bridge has two spans with the same length of 15 m. The cross-section of the bridge includes 5 $T$-shaped reinforced concrete beam. The height of the beams is 1 m and the distance between them is 1.4 m. Two abutments are spill-through ones, whereas the pier is of two-column bent.

Structural material properties are summarised in Table 1*.*

**Table 1:** Material properties.

Parameters		
Young’s modulus	Poisson’s ratio	Volumetric mass density
3.0 × ${10}^{10}$ N/${\mathrm{m}}^{2}$	0.2	2450 kg/${\mathrm{m}}^{3}$

A finite element (FE) model is generated by utilising MATLAB toolbox Stabil^22^ that is built in MATLAB software^23^. The span of the bridge is split into 30 elements using beam elements with 6 degrees of freedom at each node comprising translational and rotational displacements. FE model is then utilized to create input data (natural frequencies of the first seven modes) for the network.

The network has three layers including one input layer, one output layer, and one hidden layer. The output data represents structural damage characteristics (locations and levels). Data utilized to train, validate and test the network is randomly chosen from data sets with a rate of 70%, 15%, and 15%, respectively. The trained network is utilized to identify and quantify damages in the bridge based on regression values ($R$-values) and the Mean Squared Error (MSE). To validate the effectiveness of ANNPSOGA, ANN, PSO, and ANNPSO are also used. PSO comprises 100 populations. Social learning and cognitive learning factors are ${C}^{\mathrm{^{\prime}}}$ = 2 and ${C}^{\mathrm{^{\prime}}\mathrm{^{\prime}}}$ = 2, whereas the inertial weight parameter (${w}^{\prime}$) is 0.3. For ANN, no boundary condition is employed because this algorithm employs the GD technique to look for the best solution. 2.5% white Gaussian noise is applied to input data of ANNPSOGA, whereas only 1.5% is used to that of ANN and ANNPSO.

Damage cases are generated by decreasing the stiffness of elements. Stiffness parameters range from 0 to 1. While 1 indicates intact cases, 0 represents completely damaged cases. The data is arranged in a tabular form in which the row performs damage properties and the column performs natural frequencies. To reduce the computational cost, we only consider one beam of the bridge (other beams have the same features).

Based on the damage scenarios described above, the number of samples (${N}_{samples1}$) is calculated using Eq. (41).

41 $${N}_{samples1}={n}_{e}*{n}_{s}$$

where ${n}_{e}$ is the number of the element (${n}_{e}$ = 15 elements—with symmetric dimension; only a half of the beam is considered); ${n}_{s}$ is the number of damage scenarios (${n}_{s}$ = 50). In this case, 750 samples are used for the network.

### A free-free beam in the laboratory

**Figure 5:**
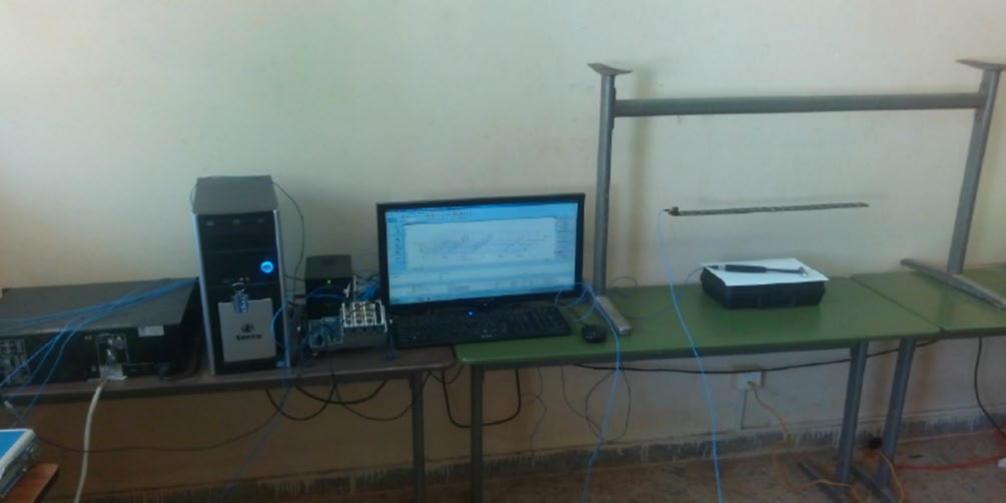
Experimental setup^24^.

A steel beam in the laboratory is utilized to assess the performance of ANNPSOGA. To diminish the impact of bearing stiffness on structural dynamic characteristics, the beam was hung on a bar described in Fig. 5. The length of the beam is 0.6 m, the width and thickness of the cross-section are 0.038 m and 0.006 m, respectively. Young's modulus of each element is selected at approximately 200 GPa (the material heterogeneity of the beam is taken into account). The Volumetric mass density and Poisson's ratio are 7850 ${\mathrm{kg}/\mathrm{m}}^{3}$ and 0.3, respectively.

To obtain the dynamic characteristics of the beam, an experimental measurement was conducted. The vibration source was generated by applying a hammer creating artificial excitation forces (Fig. 5). Accelerometers (PCB 356A15) were located at the edge of the beam to obtain structural dynamic behaviour. Figure 5 illustrates the process of making measurements of one setup.

**Figure 6:**
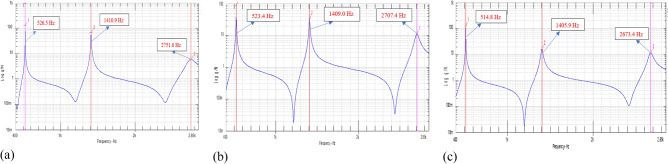
Damaged cases: (**a**) intact case; (**b**) depth of cut—3 mm; (**c**) depth of cuts—6 mm.

To extract structural dynamic behaviour, the peaking pick method was applied. The intact case was first applied. The beam was then damaged by creating cuts (3 mm, 6 mm) in the middle of the beam. The natural frequencies of all cases are shown in Fig. 6 and Table 2.

**Table 2:** Natural frequencies of the first 3 modes.

Depth of cuts (mm)	${f}_{1}$ (Hz)	${f}_{2}$ (Hz)	${f}_{3}$ (Hz)
0	526.5	1410.9	2751.6
3	523.4	1409.0	2707.4
6	514.8	1405.9	2673.4

A FE model is constructed to validate the experimental beam. The beam comprises 31 elements employing two-dimensional elements with three DOFs at each node including translational displacement in the $X, Y$—axes, and rotational displacement in the—$Z$axis. To create data for the network, the FE model of the beam has to be updated (model updating) to determine uncertain parameters. Because the free-free beam was used (the uncertainty of boundary conditions is eliminated), only the uncertainty of material properties including Young's modulus of 31 beam elements and 1 volumetric mass density are used for model updating.

To update uncertain parameters of the beam, PSO is employed. The population is 100. The cognition learning factor and social learning factor of PSO are 0.2. The inertia weight parameter of PSO (${w}^{\prime})$ is 0.3. The stop condition of the model updating process is selected based on two conditions: the deviation between calculated and desired outputs is lower than ${10}^{-6}$ or the number of iteration (100) is completed.

## Results and discussion

### Discussion about results of numerical examples

**Figure 7:**
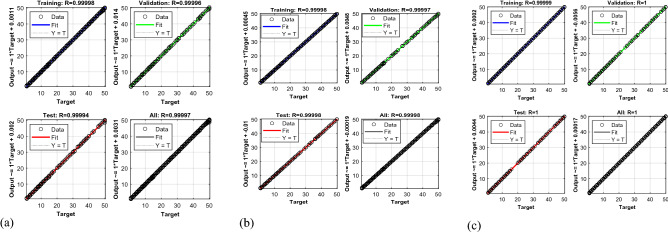
*R*-values: (**a**) ANN; (**b**) ANNPSO; (**c**) ANNPSOGA.

Figure 7 shows that regression $R$-values of the trained network. $R$-values are higher than 0.9999 and the data in the test and validation data sets are located alongside the 45-degree line (regression line). This demonstrates that a close correspondence between predicted and desired results is achieved.

**Figure 8:**
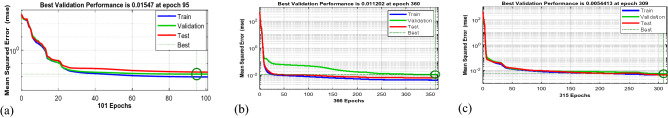
MSE-values: (**a**) ANN; (**b**) ANNPSO; (**c**) ANNPSOGA.

Figure 8 and Table 3 show that the MSE-value calculated by ANNPSOGA is the lowest, at 0.0045, whereas the MSE-values determined by ANN, ANNPSO are 0.0124, and 0.0057, respectively. Besides, the $\mathrm{R}$*-*value of ANNPSOGA surpasses those of ANNPSO, and ANN. This means that the analytical outputs (damage location and severity) determined by ANNPSOGA are close to the actual results than ANNPSO, PSO, and ANN. MSE value is determined by ANN as the highest because this algorithm applies the GD technique, which is entrapped in the local minima. ANNPSO employs PSO to identify the starting point for the network, but the network is still trapped in local minima in the process of training the network.

**Table 3:** Obtained results of methods for single damage cases.

Methods	Mean square error (MSE) values	Regression (*R*) values	Computational cost (second-s)
PSO			6109
ANN	0.0124	0.99997	14.21
ANNPSO	0.0057	0.99998	340.73
ANNPSOGA	0.0045	1	81.34

ANNPSOGA demonstrates the ability to find the optimal solution because this method contains both beneficial features of ANN and PSOGA. A clear illustration is that to remedy the shortcomings of separate ANN and metaheuristic algorithms, the capacity of each algorithm is employed at the right time during the process of the search for the best solution. Specifically, the GD technique first is applied to increase convergence speed. If the network is entrapped in concave surfaces (local minima), global search techniques are utilized to escape elements from those disadvantageous areas. This parallel combining process is reproduced till the number of iterations is reached or the target is obtained.

In terms of computational cost, ANNPSOGA requires more time than ANN, but the deviation is not too significant. PSO expends a huge amount of time on seeking the global best. Specifically, ANNPSO and ANNPSOGA, ANN spend 340.73 s, 81.34 s, and 14.21 s to determine the best solution, respectively, whereas PSO spends the most time (6109 s) because this algorithm is based on the stochastic technique in which the performance of the next step in numerous cases, is not improved compared to the previous ones.

**Figure 9:**
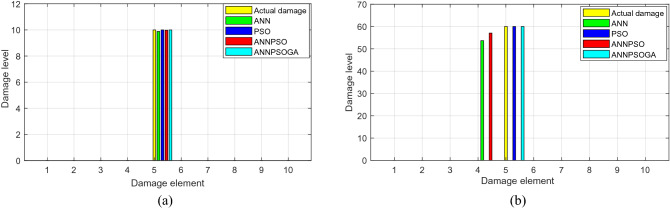
Damage identification of element 5 applying PSO, ANN, ANNPSO and ANNPSOGA: (**a**) 10% damage and (**b**) 60% damage.

Figure 9 shows that ANN and ANNPSO identify the damage location and level of trained cases inaccurately. For cases where the data is outside the trained dataset, some errors occur. For example, ANN and ANNPSO detect 53% and 57% damages occurring at element 4 (Fig. 9b). However, the real damage occurs at element 5 (60% of damage). PSO and ANNPSOGA possibly identify damages in all cases exactly.

## Discussion about results of a free-free beam in the laboratory

Table 4 shows the natural frequencies of the beam before and after model updating.

**Table 4:** Natural frequencies before and after model updating.

Modes	Before (Hz)	After (Hz)	Measurement (Hz)
1	525 (0.19%)	526.3 (0.03%)	526.5
2	1406 (0.28%)	1410.9 (0%)	1410.9
3	2743 (0.29%)	2751.6 (0%)	2751.6

After model updating, the correspondence between the simulation and the measurement is obtained. The deviation of natural frequencies between FEM and measurement is lower than 0.03%. The updated model is then utilised to generate data sets for the network. The input data consists of the first three natural frequencies, the output data (target) comprising damage locations and levels.

The network comprises one input layer, one output layer, and one hidden layer. A hidden layer with 7 neurons is selected based on optimal iteration. Data utilized to train (70%), validate (15%), and test (15%) the network is randomly chosen from data sets. The trained network is used for damage identification of the tested beam. For comparison with ANNPSOGA, ANNPSO, ANN, and PSO are also used. For PSO, the population number is 100, the values of social learning and cognitive learning factors are ${C}^{\mathrm{^{\prime}}}$= 2 and ${C}^{\mathrm{^{\prime}}\mathrm{^{\prime}}}$= 2, whereas the inertial weight parameter (${w}^{\mathrm{^{\prime}}}$) is 0.3.

Table 5 shows obtained results of methods for single damage cases.

**Table 5:** Obtained results of methods.

Methods	Mean square error (MSE) values	Regression (${\varvec{R}}$) values	Computational cost (second-s)
PSO			8594
ANN	1.0428	0.9946	168
ANNPSO	0.5612	0.9985	438
ANNPSOGA	0.4651	0.9987	195

Table 5 shows that ANNPSOGA provides a smaller deviation between the predicted and real results compared to ANNPSO, ANN, and PSO (MSE-value provided by ANNPSOGA is the lowest (0.4651) compared with ANNPSO, ANN, and PSO, at 0.5612, 1.0428, respectively). The $R$-value provided by ANNPSOGA is also higher than those of ANNPSO, and ANN. In terms of computational cost, the process of seeking the global best of PSO is most time-consuming (8594 s) compared to ANN, ANNPSOGA, and ANNPSO with 168 s, 195 s, and 438 s, respectively.

**Figure 10:**
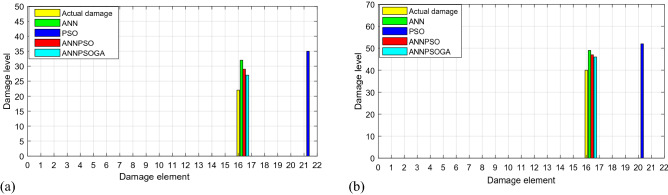
Damage identification employing ANN, PSO, ANNPSO and ANNPSOGA (**a**) 22% damage at element 16 and (**b**) 40% damage at element 16.

From Fig. 10, it can conclude that PSO fails to detect damages in the considered structure. The main reason is that PSO relies only on stochastic techniques and cannot learn from experience to improve its performance as ANN. In this case, if the target function contains too little data (in this measurement, only the first three natural frequencies are obtained), and the research space is too large (31 elements causing many the same solutions), PSO cannot determine the best solution exactly. ANN, ANNPSO, and ANNPSOGA identify damage locations exactly. The level of damage determined by ANN, ANNPSO, and ANNPSOGA is quite close to those calculated by the experimental formula Eq. (40).

## Conclusions and future works

In this work, a novel hybrid ANN is proposed to identify damages in structures. The core idea of this approach is that the stochastic search capacity of PSOGA is applied to work parallel with the GD technique to prevent the network from trapping in local minima throughout the process of training the network. ANNPSOGA extremely increases the efficiency of the conventional ANN since the fast convergence of the GD technique reduces the computational cost and the global search capacity of PSOGA not only possibly deals with local minima problems but also improves the accuracy. This solution is superior to the previous research that only applies the capacity of global search techniques to obtain beneficial starting points and to assist the network in removing local minima at the first positions. To evaluate the performance of ANNPSOGA, the numerical model and the experimental model are employed. The proposed method is also compared with the traditional ANN, ANNPSO, and PSO. Based on the obtained results, several main conclusions are drawn as follows:

- All algorithms PSO, ANN, ANNPSO, ANNPSOGA demonstrate their capacity for damage detection problems. All $R$-values are higher than 0.9 and MSE—values are small.
- Due to being based on GD technique, ANN is trapped into local minima. This leads to errors when using ANN for damage detection problems, especially if the complicated network contains too many local bests.
- PSO proves its ability when detecting the damages of numerical models accurately. However, this result is only achieved when the objective function contains sufficient data and information (occurs only in ideal conditions, when the target function can be freely chosen). Moreover, the process of seeking the best solution of PSO and other metaheuristic algorithms is time-consuming. This drawback is creating barriers to the real applications of metaheuristic algorithms when the structure is much more complicated.
- Although other hybrid ANNs are superior to traditional ANN in some cases, their outperforms are unstable and cannot thoroughly deal with local minimum problems.
- The parallel working of GD techniques and global search techniques possibly lessens the effect of noise on the regression model used for ANN.
- ANNPSOGA can deal with the problems of local minima of traditional ML as well as ANN and extremely reduces computational cost compared to other metaheuristic algorithms. Therefore, this proposed approach is highly promising to apply for real-world problems (large-scale structures with more elements and a large degree of freedom).
- Although ANNPSOGA has obvious advantages, it is commonly acknowledged that this algorithm is not as efficient as deep learning (DL) algorithms when the input is image data. The reason is that ANNPSOGA as well as other ANN models do not have the ability to self-extract data features from the convolutional class and the trained classifier at the same time.
- From obtained results, some main future works can be proposed as follows:
  - This proposed approach should be employed to detect real damages to existing structures (buildings and bridges) in further research.
  - The capacity of the global search technique will be employed to deal with local minimum problems of DL models.

## Data Availability

All data generated or analyzed during this study are included in this published article.
